# Assessment Parameters for Arrayed Pulse Wave Analysis and Application in Hypertensive Disorders

**DOI:** 10.1155/2022/6652028

**Published:** 2022-02-17

**Authors:** Zi-Juan Bi, Xing-Hua Yao, Xiao-Juan Hu, Pei Yuan, Xiao-Jing Guo, Zhi-Ling Guo, Si-Han Wang, Jun Li, Yu-Lin Shi, Jia-Cai Li, Ji Cui, Jia-Tuo Xu

**Affiliations:** ^1^Basic Medicine College, Shanghai University of Traditional Chinese Medicine, 1200 Cailun Road, Shanghai 201203, China; ^2^Shanghai Innovation Center of TCM Health Service, Shanghai University of Traditional Chinese Medicine, 1200 Cailun Road, Shanghai 201203, China

## Abstract

Study on the objectivity of pulse diagnosis is inseparable from the instruments to obtain the pulse waves. The single-pulse diagnostic instrument is relatively mature in acquiring and analysing pulse waves, but the pulse information captured by single-pulse diagnostic instrument is limited. The sensor arrays can simulate rich sense of the doctor's fingers and catch multipoint and multiparameter array signals. How to analyse the acquired array signals is still a major problem in the objective research of pulse diagnosis. The goal of this study was to establish methods for analysing arrayed pulse waves and preliminarily apply them in hypertensive disorders. While a sensor array can be used for the real-time monitoring of twelve pulse wave channels, for each subject in this study, only the pulse wave signals of the left hand at the “*guan*” location were obtained. We calculated the average pulse wave (APW) per channel over a thirty-second interval. The most representative pulse wave (MRPW) and the APW were matched by their correlation coefficient (CC). The features of the MRPW and the features that corresponded to the array pulse volume (APV) parameters were identified manually. Finally, a clinical trial was conducted to detect these feature performance indicators in patients with hypertensive disorders. The independent-samples t-tests and the Mann–Whitney *U*-tests were performed to assess the differences in these pulse parameters between the healthy and hypertensive groups. We found that the radial passage (RP) APV_*h*1_, APV_*h*3_, APV_*h*4_, APV_*h*3/*h*1_ (*P* < 0.01), and APV_*h*4/*h*1_ (*P* < 0.05) were significantly higher in the hypertensive group than in the healthy group; the intermediate passage (IP) APV_*h*4_, APV_*h*3/*h*1_ (*P* < 0.05), and APV_*h*4/*h*1_ (*P* < 0.01) and t*h*e mean APV_*h*3_, APV_*h*3/*h*1_ (*P* < 0.05), and APV_*h*4/*h*1_ (*P* < 0.01) were significantly higher in the hypertensive group than in the healthy group, and the ulnar passage (UP) APV_*h*4/*h*1_ (*P* < 0.05) was clearly elevated in the hypertensive group. These results provide a preliminary validation of this novel approach for determining the APV by arrayed pulse wave analysis. In conclusion, we identified effective indicators of hypertensive vascular function. Traditional Chinese medicine (TCM) pulses comprise multidimensional information, and a sensor array could provide a better indication of TCM pulse characteristics. In this study, the validation of the arrayed pulse wave analysis demonstrates that the APV can reliably mirror TCM pulse characteristics.

## 1. Introduction

Pulse diagnosis is one of the four diagnostic methods of traditional Chinese medicine (TCM) and has been the essence and inherited summary of TCM for thousands of years. The richness and diversity of pulses determine the difficulty of pulse diagnosis. With the development of science and technology, great progress has been achieved with the modernization of pulse diagnosis, and pulse wave signals can be converted into visual digitized pulse diagrams. The morphological characteristics of the pulse wave are indicators of blood pressure, vascular wall tension, combined force of the overall vessel displacement, and phase changes. These characteristics are closely related to cardiac systolic and diastolic functions and the state of the blood vessel wall [1]. A complete pulse beat cycle is able to reflect features of cardiac pumping, and these characteristic parameters of the pulse wave are interpreted by time-domain analysis. Thus, we can objectively determine whether there is arterial stiffness or cardiac dysfunction. Pulse waves are of great utility in the clinical diagnosis of cardiovascular disease, particularly in asymptomatic populations. These demand pulse waves have good authenticity, reliability, and accuracy. Pulse diagnosis is a method used to detect *yin* and *yang*, *interior* and *exterior*, *cold* and *heat*, *asthenia,* and *sthenia* [2]. Pulses contain a wealth of physiological information, and receptors are widely distributed in the finger pulp, which allows the identification of different pulses. Additionally, the objectivity of pulse diagnosis facilitates the evaluation of diseases.

At present, the objectivity of single-pulse diagnosis is relatively mature. However, single-pulse diagnosis cannot fully reflect the characteristics of pulses or stereoscopic sensations under finger pulsation performed by doctors. Accordingly, to better evaluate the pulse, modern technology has been applied in the research and development of pulse diagnostic instruments. Sensor arrays can be used to reflect the temporal and spatial characteristics of radial artery pulse waves, which are similar to the pulse diagnostic method used by Chinese physicians [3–5]. The principles, content, and methods of pulse diagnosis determine the utility of the sensor array. Although arrayed pulse waves can reflect pulse characteristics from different angles, the methods for analysing arrayed pulse waves are still in the early stage, which highlights the need for an arrayed pulse wave analysis method based on pulse diagnostic principles.

Capacitance pressure sensors with good repeatability and sensitivity can simulate a doctor's finger pressure well. From a bionic point of view, such sensors consist of a flexible surface for contact-based sensing and extract pulse wave data based on modern computer information processing techniques [6]. Although sensor arrays have obvious advantages in detecting pulses, these advantages are not supported by data [7]. The common pulse diagnostic methods in TCM include floating, medium, and heavy. Capacitance sensors have a wide measurement range and convenient mechanical characteristics, as well as the following advantages [4]. First, capacitance sensors can distinguish the pulse wave pressure of different individuals well. Second, capacitance sensors have a simple structure, good temperature stability, and strong adaptability, which enable pressure sensors to withstand enormous temperature changes and thereby contribute to various clinical applications. Third, capacitance sensors show a good dynamic response, which facilitates the measurement of rapidly changing parameters, such as instantaneous pressure. All of these factors are useful for detecting subtle changes in pulse waves. Capacitance sensors require minimal effect energy, which means that small and thin sensors, i.e., sensors with a very low mass, can be manufactured. This feature allows the design of multiple sensing channels and synchronous detection by multiple channels [8].

Although much work has been done in the research and development of sensor arrays, the methods for analysing arrayed pulse waves have not yet been established [9–12]. Such methods are essential for the clinical application of sensor arrays. Existing studies have shown that pulse wave abnormalities are closely associated with cardiovascular disorders, which provides strong evidence for the assessment of the physiological and pathological status of individuals [13, 14].

In this work, we synchronously monitored multichannel pulse waves by real-time visualization using a Pressure Profile System (PPS) capacitance pressure sensor. The left wrist radial artery at the “*guan*” position was chosen as the common measurement site. By calculating the average pulse wave (APW) of every channel, the most representative pulse wave (MRPW) could be matched and the array pulse volume (APV) could be manually identified from the feature points corresponding to the MRPW. The APV represents the average volume of one pulse beat per unit of time, which can be computed by a linear interpolation algorithm [15]. Here, a sensor array based on pressure was applied to achieve the multipoint monitoring of radial arterial pulse waves. Pulse diagrams collected by the sensor array have multipoint and multidimensional characteristics, which are consistent with the principle and clinical needs of TCM pulse diagnosis. As such, we analysed the arrayed pulse waves, and the results demonstrate that the APV has tremendous potential for providing early indications of cardiovascular function.

## 2. Materials and Methods

### 2.1. PPS Sensor Array

**Figure 1:**
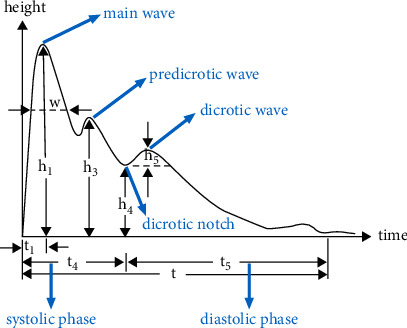
A complete pulse wave period. *h,* amplitude/height; *h*_1_, amplitude of main wave; *h*_3_, amplitude of predicrotic wave; *h*_4_, amplitude of dicrotic notch; *h*_5_, amplitude of dicrotic wave; *t*_1_, time between start point of pulse wave and main wave; *t*_4_, time between start point of pulse wave and dicrotic notch; *t*_5_, time between dicrotic notch and end point of pulse wave; *t*, time of enteric pulse wave; *w*, width of main wave in its one-third amplitude position.

**Figure 2:**
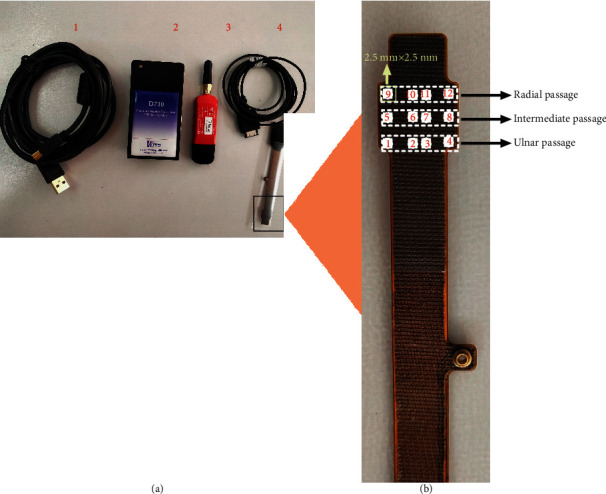
Introduction of PPS. (a) Schematic of the PPS capacitance pressure sensor. 1, USB cable for the rechargeable wireless Bluetooth interface box; 2, rechargeable wireless Bluetooth interface box; 3, Bluetooth dongle; and 4, CAPSENSE wrist module. (b) Distribution of 12 channels.

Time-domain analysis is commonly used in pulse wave analysis, and the obtained pulse wave parameters facilitate the diagnosis of cardiovascular disease [16]. A typical pulse wave (Figure 1) incorporates *h*_1_, *h*_3_, *h*_4_, *h*_5_, *t*, *t*_1_, *t*_4_, *t*_5_, and *w*. A pulse period can be divided into an ascending branch and descending branch, both of which form the main wave (*h*_1_). The valley of the descending branch is called the dicrotic notch (*h*_4_), and the peak between the main wave and dicrotic notch is the predicrotic wave (*h*_3_), which is also known as the tidal wave. The dicrotic wave (*h*_5_) follows the dicrotic notch. In this study, a PPS sensor array was provided by Pressure Profile Systems (Pressure Profile Systems, Inc., USA), including hardware and software components. The Chameleon software (Pressure Profile Systems, Inc., USA) and drivers were installed in advance. The hardware consists of four parts: a USB cable with a FingerTPS (Finger Tactile Pressure Sensing) power supply, a rechargeable wireless Bluetooth interface box, a Bluetooth dongle, and a CAPSENSE wrist module. The wrist module was placed at the “*guan*” position of the left hand. The pulse wave signal was received through the rechargeable wireless Bluetooth interface box and was transmitted to a computer. The USB cable was used to charge the interface box (Figure 2(a)).

### 2.2. APW and MRPW

The original pulse wave signal should first undergo noise removal by band-pass filtering, including high- and low-frequency noise removal. The purpose of denoising is to remove interference, such as power line interference, electromagnetic signals, baseline drift, myoelectric activity, system noise, and noise generated by body movement during the pulse wave acquisition process. Thus, the main wave is isolated as much as possible, and the other frequencies of the interfering waves are suppressed. The pulse rate is 60∼100 beats per minute, and the effective frequency ranges from 1 to 4 Hz. Since the main pulse wave occupies most of the energy in the entire pulse wave, we chose a band-pass filter of 1∼4 Hz to filter the original pulse wave. The band-pass filter was designed using MATLAB, with sixth order and two band-pass cutoff points of 1 Hz and 4 Hz using a Chebyshev I type infinite response filter [17].

**Figure 3:**
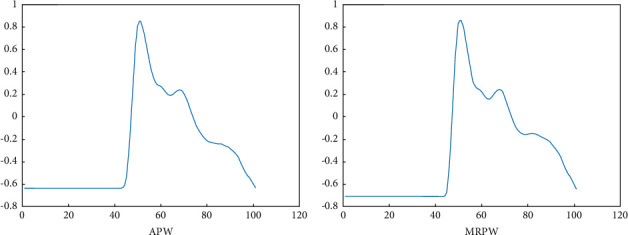
Comparison of APW and MRPW. (a) APW of a 26-year-old man. (b) MRPW of a 26-year-old man.

For the pulse wave recorded by each channel, we calculated the APW. The correlation coefficient (CC) was used to find the best-matching pulse wave (called the MRPW) with MATLAB.  Figure 3 shows the single-channel APW and MRPW of a 26-year-old man. Finally, we manually identified the characteristic parameters of the MRPW that corresponded to the APV.

### 2.3. APV

**Figure 4:**
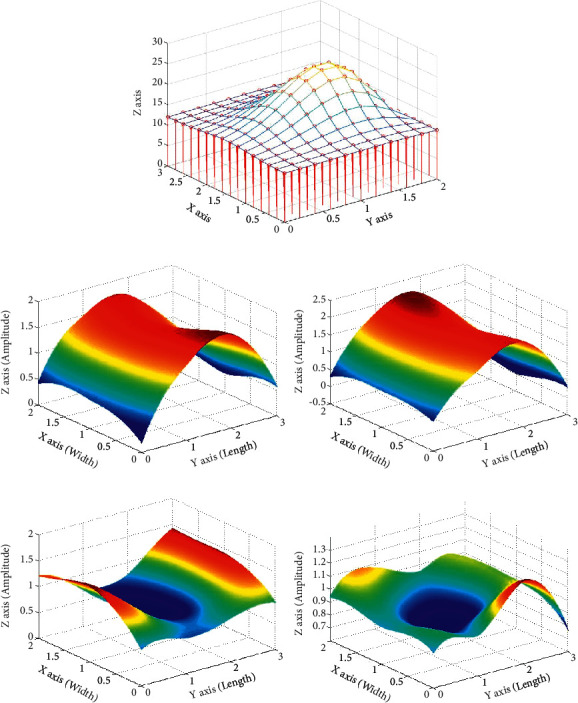
Three-dimensional pulse diagram. (a) Simulated three-dimensional pulse diagram. (b) Variation of the APV in a pulse period. 1, Radial artery vasoconstriction and increased point amplitude. 2, Expansive change in the middle when the average amplitude of 12 points reached its maximum. 3, Radial artery vasodilation and decreased point amplitude. 4, Funnel-like variation in the middle when the average amplitude of 12 points reached its minimum.

Using the PPS sensor array, we could simultaneously obtain twelve-channel pulse times, which provided twelve data points for each sampling time. Based on the position of the sensor array, the twelve points can comprise a two-dimensional array. F represents the amplitudes of the twelve points, and the F values of the 3 *∗* 4 array were interpolated into an *N* *∗* *N* array by linear interpolation. The *N* value was 1,000; i.e., the 3 *∗* 4 two-dimensional array was transformed into a 1,000 *∗* 1,000 array, *M*, containing the amplitudes of multiple points forming a surface. Thus, a three-dimensional pulse map could be constructed (Figure 4(a)). This map could simultaneously reflect the pulsation effects of vessels at different points in time and comprehensively reflect pulse characteristics. For every measurement time, the volume (*V*) was calculated using the following equation:

(1) $$\begin{array}{c} V=S*\sum_{i=1}^{N}\sum_{j=1}^{N}Mij, \end{array}$$

where *V* represents the volume at different times, and *S* is the area that is bounded by the pulse width (*x*-axis) and pulse length (*y*-axis).

Pulse wave fluctuations are periodically accompanied by energy changes. The energy of the pulse wave motion cycle of a healthy person is stable. The APV reflects the energy of each pulse cycle. We defined the APV as the average volume of one pulse beat per unit of time. A typical pulse cycle is shown in Figure 4(b). When vasoconstriction occurred, the amplitude and the average amplitude of the twelve points increased. In contrast, when vasodilation occurred, the amplitude and the average amplitude of the twelve points decreased. MATLAB was used to draw an APV graph over a pulse cycle. Since the sampling frequency was 100 Hz, the extraction frequency of the APV was also set to 100 Hz. For the determined characteristic parameters of the MRPW, the APV could be manually identified based on individual time points.

### 2.4. Experimental Protocol

Twenty-six healthy volunteers were enrolled in the experiment. To keep the baseline consistent, we recruited twenty-six age- and sex-matched patients with hypertension. Fifty-two male volunteers aged 20 to 40 years were enrolled in the clinical experiments.

### 2.5. Pulse Wave Acquisition Method

**Figure 5:**
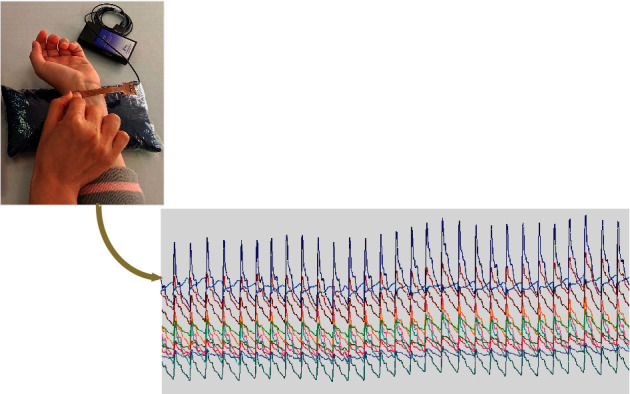
Photograph of the arrayed pulse wave signal acquisition system and a direct view of the 12-channel pulse wave.

Volunteers were required to sit quietly until their respiration, heart rate (HR), and blood pressure were stable. Blood pressure was detected by an electronic sphygmomanometer (OMRON HBP-9020, Osaka, Japan). Then, the sensor array (PPS SN7798) was fixed to the “*guan*” site of the left wrist [18] (Figure 5). Real-time pulse waves were then recorded over thirty seconds. The amplitude was recorded for three characteristic peaks and one valley in the pulse waveform. The detailed steps of the arrayed pulse wave acquisition process were as follows. (1) The pulse wave collectors and volunteers underwent standardized training. (2) Before pulse wave acquisition, every volunteer rested for 5 minutes to restore smooth breathing. The volunteers sat upright in a seat that faced the pulse wave collector. The left forearm was relaxed and stretched forward naturally with the elbow at 120 degrees. The left wrist was placed on the pulse diagnostic pillow with the palm naturally facing up. The body was straight, with steady breathing and no speech or body movement. (3) The “*guan*” position of the left hand was chosen as the pulse collection site because the strength of the wrist pulse at “*guan*” is usually stronger than that at “*cun*” or “*chi.*” At “*guan*,” the radial artery is located inside the styloid process of the radius and runs to the pulp of the middle finger. The array pulse signals from the left-hand “*guan*” position were confirmed, and the optimum pressure was determined by the general conditions for light, medium, and heavy pressure; minor adjustments were allowed as needed.

### 2.6. Statistical Analysis

Data are presented as the mean ± standard deviation (SD). SPSS 25.0 was used to statistically analyse the data. For continuous data, the independent-samples Student's *t*-tests were used to identify differences between two groups of normally distributed data, while the Mann–Whitney *U*-test was applied for nonnormally distributed data. The *P* values were two-sided, and *P* < 0.05 was considered to represent a significant difference.

## 3. Results

### 3.1. Comparison of Basic Data between the Healthy and Hypertensive Groups

As shown in Table 1, the systolic blood pressure (SBP) and diastolic blood pressure (DBP) were significantly higher in the hypertensive group than in the healthy group, as were the body weight and body mass index (BMI). The other clinical characteristics did not differ between the groups.

### 3.2. Assessment of Characteristic APV Parameters

According to the pulse diagnostic direction of the twelve single channels and the axial direction of the vessels, the twelve single channels could be divided into three-channel groups, i.e., those in the radial passage (RP), intermediate passage (IP), and ulnar passage (UP) (Figure 2(b)). For each passage, we calculated the average of the four channels; we also calculated the average of the twelve channels (mean). The APV was defined in accordance with the physiological significance of the characteristic parameters of a single-channel pulse; the details are shown in Table 2.

### 3.3. Raw and Filtered Waveforms

**Figure 6:**
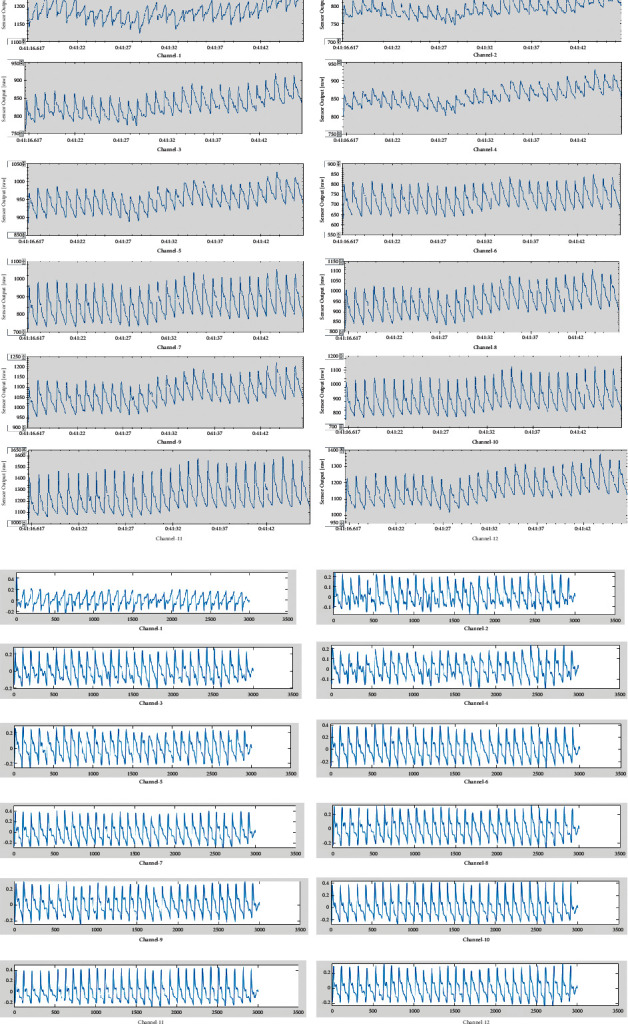
Pulse waveform in 30 seconds. (a) Raw data of 12 channels in a 26-year-old man. (b) Filtered data of 12 channels in a 26-year-old man.

In the original pulse wave, baseline drift existed due to body and wrist movement during the pulse wave acquisition process. Figures 6(a) and 6(b) show the 12-channel pulse wave signal of a 26-year-old man over thirty seconds. High-fidelity pulse wave signals of the raw data were recorded without any filtering (Figure 6(a)). After band-pass filtering, the denoised pulse wave signals exhibited better consistency (Figure 6(b)). In all the pulse wave cycles, *h*_1_, *h*_3_, *h*_4_, and *h*_5_ were visible.

### 3.4. Differences in the APV between the Healthy and Hypertensive Groups

Time-domain analysis is commonly used in pulse wave analysis and is useful for analysing two-dimensional pulse waves. Based on the characteristic parameters of a single pulse and the results of our previous study, we explored the spatial features of the APV. We calculated the absolute APV values at specific times and the relative APV ratios at different times. These indicators are important for health assessments.

**Figure 7:**
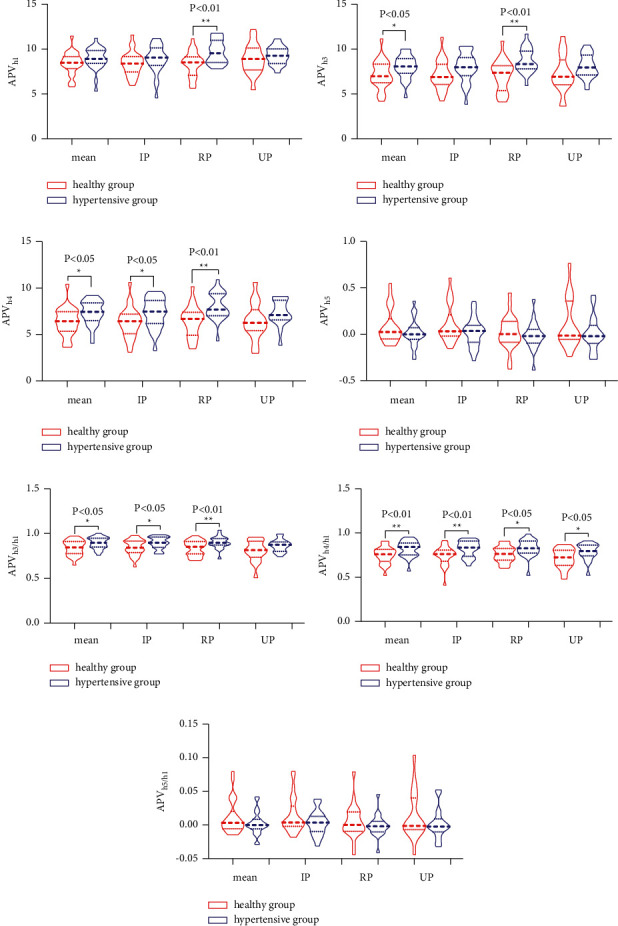
Comparison of APV parameters between the healthy and hypertensive groups. (a) APV_*h*1_. (b) APV_*h*3_. (c) APV_*h*4_. (d) APV_*h*5_. (e) APV_*h*3/*h*1_. (f) APV_*h*4/*h*1_. (g) APV_*h*5/*h*1_. RP: radial passage; IP: intermediate passage; UP: ulnar passage; mean: average of all twelve channels. ^∗^*P* < 0.05, ^∗∗^*P* < 0.01 compared with the healthy group.

The comparison of the APV between the healthy and hypertensive groups showed that the RP, IP, UP, and mean APV_*h*4/*h*1_ values were markedly higher in the hypertensive group. The RP, IP, and mean APV_*h*4_ and APV_*h*3/*h*1_ values were significantly elevated in the hypertensive group. The RP and mean APV_*h*3_ values and RP APV_*h*1_ values were also elevated in the hypertensive group, while the APV_*h*5_ and APV_*h*5/*h*1_ values showed no significant differences between the two groups (Table 3, Figure 7(a)–7(g)). These results suggested increased peripheral resistance, decreased vascular compliance, and reduced left ventricular compliance in the hypertensive group. These findings are consistent with those of an earlier study [19] and indicate that the three dimensions of the characteristic APV parameters are sensitive indicators.

## 4. Discussion

With the development of modern methods for pulse diagnosis, many high-tech materials have been applied in research on pulse diagnostic instruments. In TCM, the pulse has pulse wave characteristics and produces a global impression that is sensed by the fingers; among other information, these characteristics include pulse rate, rhythm, floating and sinking, strength and weakness, thickness and thinness, and stiffness and softness. Therefore, sensors must be as realistic as possible to detect and reflect the pulse. Single-channel pulse wave sensors are limited to the acquisition of pulse wave data, but this is essential to acquire sufficient information for disease diagnosis. Sensor arrays may compensate for this disadvantage to some extent. Different types of sensor arrays have been developed for health monitoring. Wang et al. [20] combined a pressure sensor and photoelectric sensor array that consisted of a multichannel sensor fusion structure. This pulse system was found to be effective compared with previous pulse acquisition platforms. Xu et al. [21] confirmed that a piezoelectric sensor array-based device had similar accuracy and reproducibility for measuring the pulse wave velocity (PWV). Park et al. [22] described a stretchable array of highly sensitive pressure sensors that consisted of polyaniline nanofibres and Au-coated polydimethylsiloxane micropillars that exhibited great potential in wearable devices. Sensor arrays can obtain simultaneous measurements of the width, amplitude, and other spatiotemporal parameters of dynamic pulse waves under different pressures [23].

Compared with single-channel sensors, sensor arrays may be more comprehensive in their response to pulse characteristics. PPS capacitance pressure sensors in an array can be manually pressurized and reflect the morphological characteristics of 12-channel pulse waves in real time. Single-channel pulse parameters have been proven to be effective and reliable in clinical disease studies [3, 24, 25]. For improved health monitoring, these devices have been designed to be wearable for the continuous measurement of physiological and pathological signals [26]. Although a single-channel pulse wave sensor has the advantages of flexibility and a low cost, it has limitations in extracting adequate pulse information. Sensor arrays can not only better simulate the finger pulp of a physician and reflect the rich amount of information obtained during a manual assessment but also meet the higher requirements of APW analysis.

A large amount of clinical and epidemiological evidence indicates that hypertension is a major risk factor for cardiovascular disease and that it can be characterized by impaired vascular structure and endothelial function as indicators of early vascular lesions [27]. In this study, both SBP and DBP were significantly higher in the hypertensive group than in the healthy group (Table 1). Previous studies have explained that increases in *h*_1_ and *h*_3_/*h*_1_ are associated with arterial stiffness [28]. The parameter *h*_1_ represents the ejection function of the left ventricle, which is the primary target for organ damage. In the early stage of hypertension, myocardial cells adapt to the increased workload by ventricular remodelling [29], which may lead to a compensatory increase in *h*_1_. The increased RP APV_*h*1_ in the hypertensive group in our study is consistent with the results of previous studies. The *h*_3_/*h*_1_ ratio reflects the compliance and peripheral resistance of the vessel wall [28]. Hypertension is often accompanied by arteriosclerosis [30, 31], and the PWV is increased [32], and vascular compliance is decreased in hypertension [33]. The increased RP, IP, and mean APV_*h*3/*h*1_ values in the hypertensive group in this study are consistent with the findings of previous reports [34].

Existing studies have indicated that compared with healthy populations, in hypertensive populations, the degree of arteriosclerosis is greater, the vascular elasticity is lower, and the PWV is faster [35, 36]. In time-domain pulse analyses, *h*_4_ is used to evaluate the peripheral resistance of blood vessels, and *h*_3_ is used to assess the elasticity and peripheral resistance of arterial blood vessels. In this study, APV_*h*4_, APV_*h*3_, and APV_*h*4/*h*1_ were greater in the hypertensive group; these results indicate that the peripheral resistance and vascular elasticity were elevated in the hypertensive group, which is consistent with the results of previous research.

The proposed sensor array-based pulse diagnostic method is convenient and noninvasive and can be used widely in asymptomatic individuals. This method could serve as a safe and convenient means of screening for cardiovascular disease. We found comparable results upon examining two groups of APV parameters obtained by this method for the same pathway; the IP showed more obvious differences in parameters. According to haemodynamics [37] and hydromechanics [38], blood flow can be divided into laminar and turbulent flow. The laminar flow represents ordered movement in which the flow direction of liquid per mass point is identical to the long axis of the pipe; however, the flow rate of each mass point is different. The faster the flow rate at the pipe axis, the slower the flow rate of the layer close to the pipe wall. Therefore, the difference in the average 12-channel APV between the two groups was not obvious.

Further clinical experiments need to be carried out in follow-up research. Only twenty-six subjects were included in each group for arrayed pulse wave analysis. In future research, the distribution of subjects should be expanded in terms of number, age, sex, and other health conditions. The sensor array used in this study can be used to achieve the real-time monitoring of multichannel data. The proposed array pulse analysis method primarily demonstrated that APV parameters can predict health conditions. While this equipment is currently relatively expensive, it has great potential for clinical use in pulse diagnosis, and developing an analysis method based on array pulses is essential. In this study, we proved the reliability of the APV for predicting the health status in a small cohort.

## 5. Conclusions

This study represents a new attempt to analyse arrayed pulse waves. As single-channel pulse wave sensors are limited in obtaining pulse wave information, sensor arrays can be used to help measure three-dimensional pulse waves. The sensor array area that was used in this study was very small, and subsequent sensor research will promote development in a more favourable direction. The characteristic indicator, i.e., the APV, mirrors energy changes in blood vessels. Comparison of this parameter between the healthy and hypertensive groups showed good prediction of cardiovascular function. Arrayed pulse waves still contain much information that has yet to be explored. In the future, we intend to extract multiple additional valuable characteristics from arrayed pulse waves. We plan to use different pulse wave analysis methods, including time-domain and frequency-domain analyses. Convolutional neural networks and other machine learning and deep learning methods could also be used to achieve intelligent feature point recognition and pulse classification.

## Figures and Tables

**Table 1:** Basic data between the healthy and hypertensive groups.

	Mean (SD)	
	Healthy group	Hypertensive group
Age	30.23 (5.72)	32.00 (8.34)
Height (cm)	174.27 (6.56)	174.85 (7.59)
Weight (kg)	73.08 (8.18)	80.73 (14.66)^*∗*^
BMI	24.05 (2.28)	26.35 (4.08)^*∗*^
SBP (mmHg)	124.04 (9.46)	147.70 (8.28)^*∗∗∗*^
DBP (mmHg)	76.54 (5.74)	89.50 (10.45)^*∗∗∗*^
HR (bpm)	84.62 (13.16)	83.92 (14.55)

BMI: body mass index, SBP: systolic blood pressure, DBP: diastolic blood pressure, HR: heart rate and bpm: beats per minute. ^∗^*P* < 0.05, ^∗∗∗^*P* < 0.001 in comparison with the healthy group.

**Table 2:** Definitions of characteristic APV parameters.

Parameters	Meaning
APV_*h*1_	The area under the surface formed by the set of amplitude of the main wave
APV_*h*3_	The area under the surface formed by the set of amplitude of the predicrotic wave
APV_*h*4_	The area under the surface formed by the set of amplitude of the dicrotic notch
APV_*h*5_	The area under the surface formed by the set of amplitude of the dicrotic wave
APV_*h*3/*h*1_	The area under the surface formed by the ratio of the amplitude of the predicrotic wave amplitude to the amplitude of main wave
APV_*h*4/*h*1_	The area under the surface formed by the ratio of the amplitude of the dicrotic notch amplitude to the amplitude of main wave
APV_*h*5/*h*1_	The area under the surface formed by the ratio of the amplitude of the dicrotic wave amplitude to the amplitude of main wave

**Table 3:** Comparisons of characteristic APV parameters.

Parameters		Mean (SD)		Statistics	*P* value
		Healthy group	Hypertension group		
RP	APV_*h*1_	8.623 (1.349)	9.947 (1.192)	−3.220	0.002^*∗∗*^
	APV_*h*3_	7.219 (1.784)	8.729 (1.377)	−3.470	0.001^*∗∗*^
	APV_*h*4_	6.497 (1.682)	7.940 (1.565)	−3.388	0.002^*∗∗*^
	APV_*h*5_	−0.002 (0.188)	−0.022 (0.175)	0.714	0.479
	APV_*h*3/*h*1_	0.827 (0.090)	0.874 (0.058)	−2.714	0.010^*∗*^
	APV_*h*4/*h*1_	0.743 (0.089)	0.793 (0.091)	−2.512	0.012^*∗*^
	APV_*h*5/*h*1_	0.001 (0.022)	−0.002 (0.019)	0.934	0.355

IP	APV_*h*1_	8.840 (1.387)	9.715 (1.002)	−1.326	0.191
	APV_*h*3_	7.414 (1.860)	8.617 (1.315)	−1.975	0.054
	APV_*h*4_	6.497 (2.016)	7.806 (1.404)	2.303	0.025^*∗*^
	APV_*h*5_	0.100 (0.237)	0.035 (0.183)	−1.098	0.272
	APV_*h*3/*h*1_	0.830 (0.100)	0.883 (0.055)	−2.426	0.019^*∗*^
	APV_*h*4/*h*1_	0.721 (0.128)	0.800 (0.089)	−2.739	0.009^*∗∗*^
	APV_*h*5/*h*1_	0.012 (0.029)	0.004 (0.019)	−1.226	0.220

UP	APV_*h*1_	8.643 (1.760)	9.428 (1.037)	−0.918	0.365
	APV_*h*3_	6.974 (2.203)	8.182 (1.440)	−1.410	0.168
	APV_*h*4_	6.229 (2.084)	7.360 (1.520)	−1.586	0.122
	APV_*h*5_	0.100 (0.287)	0.016 (0.182)	−0.924	0.355
	APV_*h*3/*h*1_	0.795 (0.126)	0.863 (0.074)	−1.849	0.073
	APV_*h*4/*h*1_	0.707 (0.120)	0.774 (0.100)	−2.042	0.049^*∗*^
	APV_*h*5/*h*1_	0.127 (0.039)	0.003 (0.022)	0.409	0.409

Mean	APV_*h*1_	8.739 (1.213)	9.646 (0.763)	−1.558	0.126
	APV_*h*3_	7.275 (1.751)	8.460 (1.135)	−2.228	0.030^*∗*^
	APV_*h*4_	6.454 (1.764)	7.653 (1.273)	−2.537	0.014^*∗*^
	APV_*h*5_	0.066 (0.216)	0.018 (0.160)	−1.226	0.220
	APV_*h*3/*h*1_	0.823 (0.096)	0.874 (0.062)	−2.604	0.012^*∗*^
	APV_*h*4/*h*1_	0.727 (0.106)	0.789 (0.087)	−2.836	0.007^*∗∗*^
	APV_*h*5/*h*1_	0.009 (0.026)	0.002 (0.017)	−1.098	0.272

RP: radial passage, IP: intermediate passage, UP: ulnar passage, and mean: average of twelve channels. ^∗^*P* < 0.05, ^∗∗^*P* < 0.01 in comparison with the healthy group.

## Data Availability

The datasets generated and analysed during this study are not publicly available due to data confidentiality considerations, which are an important component of the National Key Technology R&D Program of the 13th Five-Year Plan (No. 2017YFC1703301) in China but are available from the corresponding author upon reasonable request.

## Abbreviations

TCM: Traditional Chinese medicine
PPS: Pressure Profile System
APW: Average pulse wave
MRPW: Most representative pulse wave
APV: Array pulse volume
CC: Correlation coefficient
HR: Heart rate
SBP: Systolic blood pressure
DBP: Diastolic blood pressure
BMI: Body mass index
RP: Radial passage
IP: Intermediate passage
UP: Ulnar passage
PWV: Pulse wave velocity.

## Supplementary Materials

Supplementary Materials Support information—detailed instructions for the use of PPS hardware and its parameters.
