# Supplementary material for: Assessment Parameters for Arrayed Pulse Wave Analysis and Application in Hypertensive Disorders
Source: Evid Based Complement Alternat Med. 2022 Feb 17;2022:6652028. doi: 10.1155/2022/6652028 (PMC8872656; doi:10.1155/2022/6652028)
Supplement: Supplementary Materials — Support information—detailed instructions for the use of PPS hardware and its parameters. [file 6652028.f1.docx]

**Support information**

*Detailed instructions for the use of PPS hardware and its parameters are as follows:*

Before the Chameleon software is started, the battery needs to be fully charged and connected through the USB interface to a computer. The hardware components should be connected in the following sequence. First, the Bluetooth dongle is plugged into an open USB port of the computer. Second, the FingerTPS power switch is turned on prior to launching the software. When the power supply is initialized, the LED should blink green, indicating that the software is ready to connect; otherwise, the batteries still need to be charged. The batteries can be used continuously for four hours and require two to three hours to be fully charged. Finally, the Chameleon software is ready to be launched. Detailed information and an [illustrative diagram](https://www.youdao.com/w/illustrative%20diagram/#keyfrom=E2Ctranslation) of the PPS are shown in Support information Table 1 and manuscript body Figure 2b. The maximum pressure of the sensor is 300 mmHg. If this maximum range is exceeded, the sensor cannot be restored to the calibration value, which will eventually affect the measurement results. The minimum external pressure that can be detected by the sensor is 0.1 lbs (i.e., 0.045 kg). Normal room temperature and humidity have no effect on the sensor measurement.

Table 1 Performance introduction of PPS

| Array size | 10 mm×7.5 mm (4×3 grid) |
| --- | --- |
| Element size | 2.5 mm×2.5 mm |
| Thickness | ~0.5 mm |
| Full scan range | 300 mmHg |
| Scan rate | 95~100 Hz |
| Temperature range | -20~+100℃ |
| Power | USB bus power or rechargeable li |
